# First Results of Pediatric Robotic Inguinal Hernia Repair with the Senhance^®^ Surgical System: A Matched Cohort Study

**DOI:** 10.3390/healthcare12171703

**Published:** 2024-08-26

**Authors:** Roxanne Eurlings, Rianne E. M. Killaars, Hamit Cakir, Marc Dirix, Olivier Theeuws, Ludger Staib, Dietmar Stephan, Ruben G. J. Visschers, Wim G. van Gemert

**Affiliations:** 1 Department of Pediatric Surgery, MosaKids Children’s Hospital, Maastricht University Medical Center+ (MUMC+), P. Debyelaan 25, 6229 HX Maastricht, The Netherlands; 2 Faculty of Health, Medicine and Life Sciences FHML, NUTRIM School for Nutrition and Translational Research in Metabolism, Maastricht University, Universiteitssingel 40, 6229 ER Maastricht, The Netherlands; 3 European Consortium of Pediatric Surgery (MUMC+, Uniklinik Aachen, Centre Hospitalier Chrétien Liège), Maastricht, P. Debyelaan 25, 6229 HX Maastricht, The Netherlands; 4 Department of General and Visceral Surgery, Klinikum Esslingen, Hirschlandstraße 97, 73730 Esslingen am Neckar, Germany; 5 Department of General and Visceral Surgery, Marienkrankenhaus Siegen, Kampenstraße 51, 57072 Siegen, Germany

**Keywords:** pediatric inguinal hernia, laparoscopic hernia repair, robot-assisted hernia repair, Senhance^®^ Surgical System

## Abstract

Introduction: Inguinal hernia repair (IHR) is one of the most common procedures in pediatric surgery. In children, the application of robotic surgery is limited, meaning safety and efficacy is still to be assessed. This report is the first one worldwide that describes inguinal hernia repair in children using the Senhance^®^ Surgical System (SSS^®^). The aim of this matched cohort study is to assess safety and feasibility of robot-assisted IHR (RIHR) in children, compared to conventional laparoscopic IHR (LIHR). Patients and methods: This pilot study included 26 consecutive patients between 3 months and 8 years old who underwent RIHR (31 IH’s) with the SSS^®^ between 2020 and 2024. These cases were matched based on gender, age, and unilateral or bilateral IH, with 26 patients (32 IH’s) who underwent conventional LIHR. Results: There was a significant difference in total anesthesia time, which is most likely due to the extra time needed to dock the robot in the RIHR cases. No significant difference was seen in surgical time. One recurrence (3.2%) was diagnosed in both groups. One patient in the LIHR group was readmitted on the day of discharge due to a hemorrhage. No intervention was necessary, and the patient was discharged 1 day later. Discussion: In this pilot study, the use of the robotic system was safe and feasible. More experience, further improvement of the system for use in very small children, and investigation in a larger sample size with long-term follow-up is necessary to evaluate efficacy.

## 1. Introduction

With a prevalence of inguinal hernia (IH) in children of 1–5% in all newborns and as high as 9–11% in prematurely born babies, repair of the IH is one of the most commonly performed procedures in pediatric surgery [1,2,3,4]. Laparoscopic inguinal hernia repair (LIHR) has become increasingly more popular. The laparoscopic approach offers various advantages, such as faster recovery, less postoperative pain, the possibility of exploration of the contralateral side, and better cosmetic results [5,6,7]. Several laparoscopic techniques in children have been described, either using a trans-peritoneal or pre-peritoneal approach [8]. The trans-peritoneal intra-corporal approach is performed by partial resection of the hernial sac, followed by closure of the internal inguinal ring with a purse string suture or an N-suture, both of which have similar long-term outcomes. As minimal invasive surgery evolves, there is also more interest in a robotic approach. In adults, there is already extensive experience with robotic IHR, showing the robotic approach is safe and effective, with minimal complications [9]. In children, however, experience with robotic surgery is limited and the operation technique is completely different compared to IHR in adults. In November 2020, the first procedures with the Senhance^®^ Surgical System (SSS^®^) were performed in the Maastricht University Medical Center (MUMC+).

To our knowledge, this is the first report describing IHR in children using the SSS^®^. The aim of this pilot study is to assess the safety and feasibility of robot-assisted IHR (RIHR) in children, compared to the conventional laparoscopic IHR (LIHR), since the operative technique is the same. We also wanted to share our lessons learned and formulate recommendations for the use of the SSS^®^ in children based on our first experience (lessons learned).

## 2. Materials and Methods

### 2.1. System

The SSS^®^ (Asensus Surgical Inc., Italia S.R.L. (Milan, Italy)) was installed in the MUMC+ in September 2020. All involved healthcare providers (pediatric surgeons and OR nurses) were trained in a dry lab and on human cadavers to work correctly with the system. For the first 10 procedures, a technical specialist and a senior proctor surgeon (D.S.) were present in the OR.

The SSS^®^ is designed to assist in accurate control of laparoscopic and thoracoscopic surgery. The system offers six degrees of freedom if the 5 mm and 10 mm articulated instruments are used and five degrees of freedom when using the 3 mm instruments, since they are too small for articulation [10]. The system received a CE-marking for treatment of pediatric patients with a weight of at least 10 kg. The system consists of a cockpit, which is the open console where the surgeon operates, the manipulator arms (three or four), and a node, which is a relay connecting the cockpit input to the manipulator arms and transmits the video signals to the monitor.

### 2.2. Patients

This pilot study was approved by the Institutional Review Board (or Ethics Committee) of Maastricht (2020–2329) on 1 December 2020 as a study involving humans. All pediatric patients (younger than 16 years) with an IH who were operated with the SSS^®^ in the period from September 2020 and February 2024 were included in this report. Exclusion criteria were combined surgical procedure, the presence of any relevant severe condition that, in the opinion of the investigator, may interfere with the participation in the study, and parents or children with insufficient understanding of the local language. The decision to propose surgery using the SSS^®^ was made by the surgeon based on the inclusion and exclusion criteria, and the majority complied with the Senhance^®^ guidelines (intended use). All parents (and patients, if age appropriate) signed an informed consent form prior to the surgery. Seven patients under 10 kg (off-label use, range 3.2–8.3 kg) were also included in this series, for which the parents also gave their written consent. Experienced colleagues and the clinical specialist of Asensus Surgical Inc. were consulted on the robotic set-up and the steps of the procedure.

For this cohort, IH was chosen because the incidence is high and the procedure is very well-known. This allows for easy implementation of new techniques.

The matched cohort was created based on the general pediatric surgery database from the MUMC+, containing all performed procedures. Subjects from the RIHR group were matched in a 1:1 ratio based on procedure, gender, age (with an accepted range of 2.5 years), and unilateral or bilateral IH. For the matched cohort, patients were considered if they underwent standard LIHR with purse string suture. Patients undergoing LIHR in the same time period (September 2020 to February 2024) as the RIHR were prioritized. Reasons for exclusion from the matched cohort were IHR using another technique, combined surgeries, or surgery of a recurrent IH.

### 2.3. Procedure and Team

The Senhance^®^ surgical system was used with three arms, of which one arm is used for a 0° or 30° 5mm camera. Patients were operated in a supine position; no prophylactic antibiotics were administered. In all patients, the contralateral inguinal side was explored to check for inguinal hernia or persistent processus vaginalis. If present, a contralateral hernia was repaired during the same procedure. Partial resection of the hernial sac and closure of the inguinal herniation with an intra-corporal purse string suture was performed in all patients using a Vicryl suture. The suture was introduced percutaneously when using 3 mm trocar. In case of 5 mm trocars and a thick subcutaneous layer, the suture was inserted through the trocar. All RIHR procedures were performed by pediatric surgeons with extensive experience with laparoscopy. In total, five pediatric surgeons alternated to perform the RIHR procedures.

The LIHR was performed with the same technique, the same materials, and by the same pediatric surgeons. In both groups, as per standard of care protocol for our facility, prematurely born children who are younger than 60 weeks post conception or children born a term who are younger than 3 months were admitted for observation for 24 h after surgery, to be monitored for possible apnea. 

### 2.4. Clinical Outcomes

Preoperative, perioperative, and postoperative data were collected from the patient’s medical records for both the RIHR and the LIHR group, e.g., baseline characteristics, day of discharge after procedure, readmissions within 30 days after surgery, etc. During the RIHR procedure, data about perioperative course, performance, operative times, and complications with the SSS^®^ were collected by a researcher. The operative times included the docking time of the robot (time to position the arms of the SSS^®^ correctly before the procedure is started), net-surgical time (time from end of docking of the robotic system to the incisions being fully closed), and total anesthesia time, corrected for bilateral procedures. 

### 2.5. Data Analysis and Statistics

For the patient demographics, descriptive statistics were used. To test the different variables for normality, the Shapiro-Wilk test was performed, and the histograms and Q-Q plots were visually inspected. The focus of the analysis was to compare all the outcomes between both surgical techniques. If normality could be assumed, the paired t-test was used to compare continuous variable; in case normality could not be assumed, the Wilcoxon Signed Rank test was used. For categorical variables, the McNemar test was performed. The analysis was performed in an intention-to-treat manner. The tests were considered statistically significant if the *p*-value was <0.05. There were two patients with missing data regarding the docking times of the robotic system. These data were not imputed. There were no other missing data. Statistical analysis was performed with the IBM SPSS Statistics for Windows, version 28.0 (IBM Corp., Armonk, NY, USA) software.

## 3. Results

### 3.1. Population Characteristics

In total, 26 consecutive patients under the age of 16 underwent RIHR with the SSS^®^ in the MUMC+. All parents gave written consent for surgery with the SSS^®^, and all patients are included in this report. The patients in the RIHR group were matched with 26 patients who underwent LIHR. Baseline characteristics are listed in Table 1. None of these baseline characteristics was significantly different. In total, 31 IH’s were operated with the SSS^®^ (one side of two bilateral cases was repaired with conventional laparoscopy) and 32 IH’s in the LIHR group.

### 3.2. Perioperative Outcomes

There was a significant difference in total anesthesia time between both groups (106.6 + 23.8 min for RIHR vs. 71.0 + 16.9 min for LIHR, *p* < 0.001), which included the 7.7 + 5.0 min mean docking time of the robotic system in the RIHR group. The net-surgical time was 50.8 + 17.0 min for the RIHR group, compared to 43.3 + 21.4 min for the LIHR group, which was not significantly different (*p* = 0.214) (Table 2). A subanalysis with ANOVA comparing the net surgical time and total anesthesia time in terms of the patient’s weight (comparing three groups: <10 kg, <20 kg and <30 kg) did not reveal a significant difference between the three groups (Total anesthesia time, corrected for bilateral procedures: *p* = 0.769, NET surgical time: *p* = 0.686). The weight for the robotic group ranged from 3.2 to 27.0 kg. There was also no significant difference in total anesthesia time, corrected for bilateral procedures (*p* = 0.706) or for net-surgical time (*p* = 0.462) when comparing the times for male patients to female patients.

### 3.3. Postoperative Outcomes

No in-hospital postoperative complications occurred in either group. All patients in both groups could be discharged from the hospital conform standard of care protocol on the day of surgery or the day after. Of the 26 patients who were operated on with the robotic system, five stayed one night. No patient stayed longer than one night. In the LIHR group, one patient had to be readmitted on the day of discharge due to a hemorrhage with a decrease in hemoglobin levels. However, the bleed stopped spontaneously, no re-intervention was needed, and the patient could leave the hospital the day after readmission. 

Recurrence rate in the RIHR group was 3.2% (one IH out of 31 RIHR) compared to 3.1% (one IH out of 32 LIHR) in the LIHR group. This was not significantly different (*p* = 1.000). Apart from the hemorrhage mentioned above, no other postoperative complications (such as vas deferens injury, vessel trauma, and surgical site infections) occurred in either group. The length of follow-up ranged from a minimum of 1 month in both groups to 3.3 years and 6.3 years in the RIHR group and LIHR group, respectively (Table 2).

## 4. Discussion

To our knowledge, this is the first report of the use of the SSS^®^ for IHR in children. Previous reports describe robotic-assisted repair of pediatric inguinal hernia with the use of the DaVinci^®^ robotic platform, mostly in older children (>16 years) [11,12,13,14,15]. However, the DaVinci^®^ platform only offers trocars/instruments of 8 mm, meaning the application in small cavities, i.e., smaller children, is limited [16]. The SSS^®^ also offers 3 mm instruments, making robot-assisted surgery available for very small children [16]. In this pilot study, no significant differences were observed in postoperative outcomes (recurrence, readmission within 30 days, and other complications, such as vas deferens injury, vessel trauma, and surgical site infections) and the recurrence rate of 3.2% with the SSS^®^ is comparable to the recurrence rates described in literature for conventional laparoscopy ranging from 0–5.7% [17]. The only significant difference between RIHR and LIHR is the total anesthesia time, which is most likely due to the fact that extra operating time was needed for docking of the robotic system. Also, there was one case where a defect instrument was encountered at the start of the surgery with the SSS^®^, leading to a waiting time before the first incision could be made. Furthermore, the learning curve of the robotic system was mainly in the docking, thus setting up the arms and the placement of the trocars in the best possible location. There was no learning curve in the actual procedure (thus net-surgical time) as this was exactly the same as the conventional laparoscopic procedure and all surgeons performing the robot-assisted surgery had extensive laparoscopic experience. No significant difference between RIHR and LIHR was observed in net-surgical time.

Robot-assisted laparoscopy offers several potential advantages compared to conventional laparoscopic surgery. One is tremor filtration, which is especially beneficial in small fields under high magnification, as is the case in small children [18,19]. The unique capability of instruments with haptic sensing ensures gentle handling of tissues and forced feedback prevents the surgeon from accidental damage to surrounding tissues, as being one of the most common complications associated with robotic surgery [20,21]. Furthermore, indexing allows the surgeon to continue moving an instrument beyond the normal range of their arms. This is facilitated by releasing a foot pedal before returning the hand controls to a comfortable position, while the instruments are kept in place. Repressing the foot pedal links the hand controls again to the instruments, allowing the surgeon to continue the previous actions [18,19]. A specific advantage of the Senhance^®^ robotic system is the guidance of camera movement by infrared eye tracking. The above-mentioned advantages of a robotic system, and the fact that the surgeon sits in an ergonomically corrected chair in a well-designed open workstation, optimizes the surgeon’s posture, ensuring minimal physical strain and fatigue [18,19]. In addition, the open module set-up of the Senhance^®^ surgical system facilitates an optimal view on the patient, system set-up, and the surgical team at the operating table, which helps with effective communication between the operating surgeon and the rest of the team. The fact that the robotic arms are separate modules allows for more freedom in placement of the trocars and in that way, easy and quick conversion to conventional laparoscopy is easy and fast. Furthermore, there is enough space around the operating table to facilitate hybrid procedures. Besides the “mechanical” advantages, the real benefit of robotic surgery is the implementation of augmented intelligence (AI). Senhance^®^ is one of the pioneers in this field, e.g., AI-controlled camera movement (follow instrument, auto zoom, etc.), 2D and 3D measurements, and digital tags. In a survey among 117 pediatric surgeons, more than half believe that there is a role for robotic surgery in children in the future [22].

**Figure 1:**
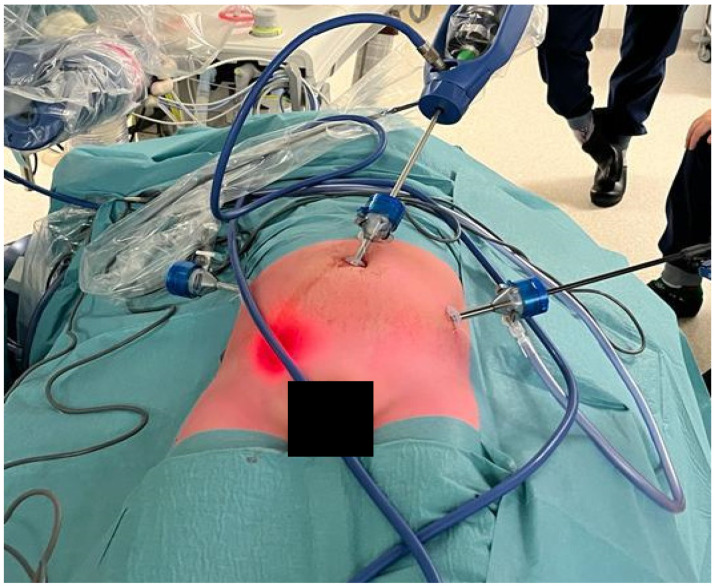
Placement of the robotic arms with the camera infra umbilical. The incision for the trocars for laparoscopic instruments was changed over time from a medial to a more lateral position, in order to provide more freedom for the surgeon to maneuver the instruments and avoid interference of the arms.

**Figure 2:**
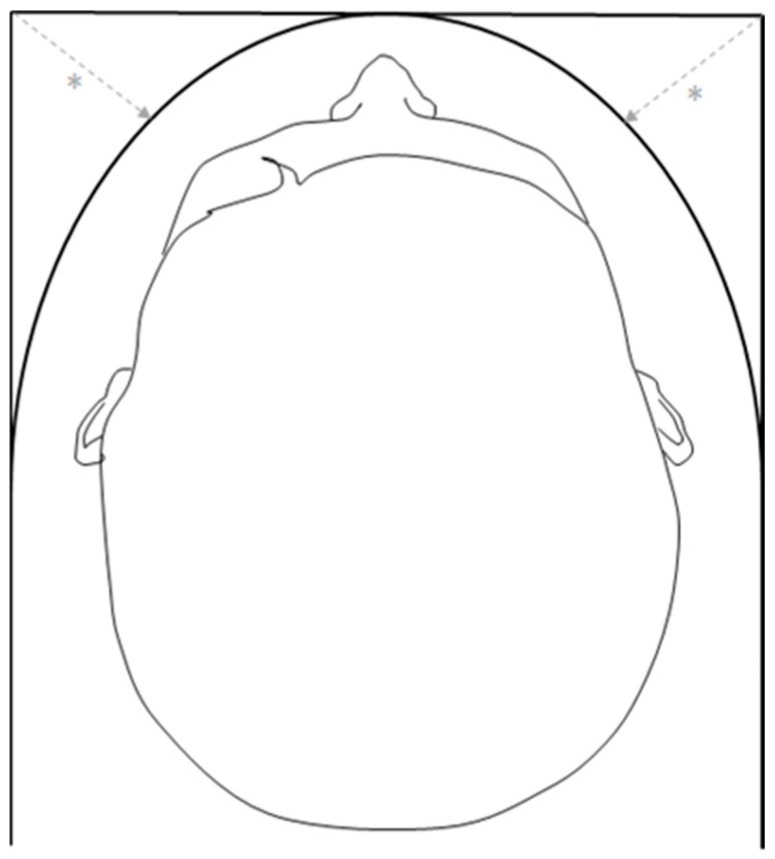
Convex arc over the patient’s head. Replacing the square arc over the top of the patient with a convex arc creates more space for the robotic arms (*), also making sure the arms do not interfere with the arc.

The limited number of procedures done in our center is too small to draw a proper conclusion regarding the learning curve. However, we do not expect a learning curve from the procedure itself because it is the same as the conventional laparoscopic procedure. In our experience, the most important learning is related to the robotic system itself, i.e., optimal positioning of the patients and robotic arms, and placement of the trocars related to body size and proportions at a particular age. These are crucial for successful application of the surgical robot. In this regard, some alterations were made in the robotic set-up to improve the robotic operating process. We found that placing the two trocars for instruments more laterally (Figure 1) and with enough distance between the trocars perpendicular to the working direction decreased the risk of interference of the robotic arms and improved the maneuverability without exceeding limits and diminishes the collision risk. Furthermore, we placed a roll under the thoraco-lumbar spine of the patient, to ensure a better angulation of the abdomen, creating better access to the inguinal region. Especially for the 3 mm instruments, the trocars must be placed as far as possible from the operating field, thereby, decreasing the momentum on the abdominal wall and minimizing the risk of bowing of the instrument. A convex arc was placed over the head of the patient instead of a square arc. This was done to facilitate more space for the robotic arms while still protecting the head when used near the head of the patient and to ensure no interference of the arms with the arc (Figure 2). 

A cost analysis was outside the scope of this pilot study, looking at safety and feasibility, but a cost analysis is currently being conducted in the MUMC+ comparing the costs of different surgical techniques for IHR in children.

There are several limitations to this study. The sample size of the groups is very small, limiting the validity of conclusions drawn from the statistical comparison. Furthermore, the LIHR group data were obtained retrospectively from the electronic patient files. In addition, the procedures were performed by five different surgeons, leading to heterogeneity among the data. In the future, a prospective, qualitative study, with a larger sample size, comparing both procedures, is necessary to confirm the results from this matched cohort.

## 5. Conclusions

This pilot study investigating RIHR with the SSS^®^ shows the procedure is safe and feasible. There is no difference in recurrence rate, postoperative complications, and net-surgical time when comparing RIHR to LIHR in our small sample population. Because of the limited number of included patients, it is not possible yet to draw valid conclusions about the efficacy of the procedure. Based on our experience, we give some recommendation for the use of the SSS^®^ in pediatric IHR. Further evaluation, especially prospectively, compared to conventional laparoscopic surgery, with a larger sample size, is necessary to determine if robot-assisted IHR can become a more prominent technique in the field of pediatric surgery. We expect that in the future, robot-assisted IHR in children will improve further because of growing experience and further development of the robotic system and might be advantageous over conventional laparoscopy.

## Figures and Tables

**Table 1:** Baseline characteristics of the patients who underwent inguinal hernia repair using the Senhance^®^ Surgical System compared to the patients of the matched cohort who underwent conventional laparoscopic inguinal hernia repair.

	Robotic IHR (n = 26)	Laparoscopic IHR (n = 26)	*p*-Value
**Age at time of surgery** (years) Mean ± SD (range)	3.0 ± 2.4 (0.25–8.0)	3.2 ± 2.7 (0.25–7.0)	0.206
**Gender**	69% male (18 male, 8 female)	69% male (18 male, 8 female)	1.000
**Weight** (kg) Mean ± SD (range)	15.1 ± 6.7 (3.2–27.0)	15.7 ± 8.2 (3.7–32.4)	0.276
**Preoperative indication**	2 bilateral IH (8%) 24 unilateral IH (92%)	2 bilateral IH (8%) 24 unilateral IH (92%)	
**Perioperative diagnosis**	7 bilateral IH (27%) 19 unilateral IH (73%)	6 bilateral IH (23%) 20 unilateral IH (77%)	

Depending on normality of the data, paired *t*-test or Wilcoxon Signed Rank test was used to assess the difference between both groups for continuous variables and McNemar test for categorical variables. Statistically significant difference was assumed if *p* < 0.05. Abbreviations: IHR, inguinal hernia repair; IH, inguinal hernia.

**Table 2:** Perioperative and postoperative outcomes of the patients who underwent inguinal hernia repair using the Senhance^®^ Surgical System compared to the patients of the matched cohort who underwent conventional laparoscopic inguinal hernia repair.

	Robotic IHR (n = 26)	Laparoscopic IHR (n = 26)	*p*-Value
Perioperative outcomes			
**Total anesthesia time, corrected for bilateral cases ^1^** (min) Mean ± SD (range)	112.2 ± 19.5 (58.0–143.0)	71.0 ± 16.9 (45.5–102.0)	<0.001 *
**Net-surgical time ^2^** (min) Mean ± SD (range)	50.8 ± 17.0 (28.0–87.0)	43.4 ± 21.4 (16.0–104.0)	0.214
**Docking time of SSS^® 3^** (min) Mean ± SD (range)	7.7 ± 5.0 (3.0–21.0)	n.a.	
**Conversion to conventional laparoscopy** (n (%))	2 (6.0) (one side of two bilateral cases)	n.a.	
**Conversion to open procedure** (n (%))	0 (0)	0 (0)	
Postoperative outcomes			
**Postoperative hospital stay** (nights) Mean ± SD (range)	0.2 ± 0.4 (0.0–1.0)	0.2 ± 0.4 (0.0–1.0)	0.739
**Readmission within 30 days** (n (%))	0 (0)	1 (3.8)	1.000
**Recurrence** (n (%))	1 (3.2)	1 (3.1)	1.000
**Other complications** (n (%))	0 (0)	1 (3.8) Same case as readmission	1.000
**Length of follow-up** (years) Mean ± SD (range)	1.6 ± 1.3 (0.08–3.3)	2.1 ± 1.8 (0.1–6.3)	0.253

Depending on normality of the data, paired t-test or Wilcoxon Signed Rank test was used to assess the difference between both groups for continuous variables and McNemar test for categorical variables. Statistically significant difference was assumed if *p* < 0.05. Abbreviations: IHR, inguinal hernia repair; SSS^®^, Senhance Surgical System^®^; n.a., not applicable. * Statistically significant difference. ^1^ Correction for bilateral procedures was done by dividing the net-surgical time of bilateral procedures by two and subtracting this time from the total anesthesia time. ^2^ Time from end of docking of the robotic system to the incisions being fully closed. ^3^ Time to position the arms of the SSS^®^ correctly before the procedure is started.

## Data Availability

All data are stored securely at the Department of Pediatric Surgery of MosaKids Children’s Hospital/Maastricht University Medical Center+ (MUMC+) without patient identifiers and is available for inspection upon request.

## Abbreviations

IH = Inguinal hernia, IHR = Inguinal hernia repair, RIHR = robot-assisted inguinal hernia repair, LIHR = laparoscopic inguinal hernia repair, SSS^®^ = Senhance ^®^ Surgical System.
